# Guidelines for Using Tele-Technology to Deliver Mind-Body Interventions for People with Mild Cognitive Impairment

**DOI:** 10.1093/geroni/igaa057.3245

**Published:** 2020-12-16

**Authors:** Kara Cohen, Tracy Mitzner, Patricia Griffiths

**Affiliations:** Georgia Institute of Technology, Atlanta, Georgia, United States

## Abstract

Individuals with Mild Cognitive Impairment (MCI) may have limited access to intervention programs that support their mental and physical health. The COVID-19 pandemic has put them at an even greater risk of not having access to such programs. While there is currently no cure, there is growing evidence that intervention programs may attenuate the progression from MCI to dementia, particularly those which 1) have potential to reduce the level of cardiovascular risk factors, 2) employ cognitively stimulating activities, and 3) create opportunities for social interaction (Petersen, Lopez, Armstrong et al., 2018; Wayne, Yeh, & Mehta, 2018; Mortimer, Ding, Borenstein et al., 2012). Many mind-body interventions, such as tai chi, yoga, and mindfulness classes, contain these three elements and have been shown to benefit individuals diagnosed with MCI, including improving cognition (e.g., Wells, Kerr, Wolkin, et al. 2013; Yang, 2016). Tele-technology (i.e., technology that supports communication between people who are not co-located) can aid in overcoming the logistical barriers by bringing instructors and interventions to these individuals to help them stay engaged and attend activities more frequently from the comfort and convenience of their home. We will present recent findings from a user study with 8 stakeholders (4 subject matter experts, 2 individuals with MCI, 2 care partners) to assess barriers and facilitators to using tele-technology to bring instruction of mind-body interventions to individuals diagnosed with MCI. This poster will present guidelines for delivering such interventions based on our findings from the user study, including safety and training protocols.

